# The Royal College of Physicians on Small Pox and Vaccination

**Published:** 1871-04

**Authors:** 


THE ROYAL COLLEGE OF PHYSICIANS ON SMALL-POX AND VACCINATION.—The Royal College of Physicians have considered it their duty to issue at the present time, when small-pox prevails extensively as an epidemic, and when much anxiety exists in the public mind respecting it, a document regarding small-pox, and the protective influence of vaccination and re-vaccination. With regard to the advantages to be derived from re-vaccination the College states that these "may be best secured, both for the community and for the individual, by a systematic performance of the operation on every person upon passing the age of puberty. For the community, because a well-grounded confidence would replace the present recurring panics about small-pox; for the individual, because the operation performed as part of a system would be done in the manner most certain to be efficient, and not, as at present too often happens, under conditions little conducive to a protective result. The practice of repeated or periodic revaccination does not appear to be generally necessary. But in instances where a person, after re-vaccination, has been subjected to serious constitutional or climatic changes, and is subsequently more than ordinarily exposed to the infection of small pox, a further revaccination may properly be advised."

